# Supplementary material for: Predicting visual function by interpreting a neuronal wiring diagram
Source: Nature. 2024 Oct 2;634(8032):113–23. doi: 10.1038/s41586-024-07953-5 (PMC11446822; doi:10.1038/s41586-024-07953-5)
Supplement: Supplementary file 6 — CRF and ERF predictions for individual TmY4 and TmY9 cells. Analogous to Supplementary Data 3, but for TmY target types. Shown are the top four monosynaptic pathways, the strongest pathway passing through each of the top ten intermediary types (ranking from Extended Data Fig. 7), and the trisynaptic pathway Tm1–TmY–Dm3–TmY (see the section entitled Prediction of spatial normalization). [file 41586_2024_7953_MOESM6_ESM.zip › DataS4/TmY9q/720575940627193469.pdf]

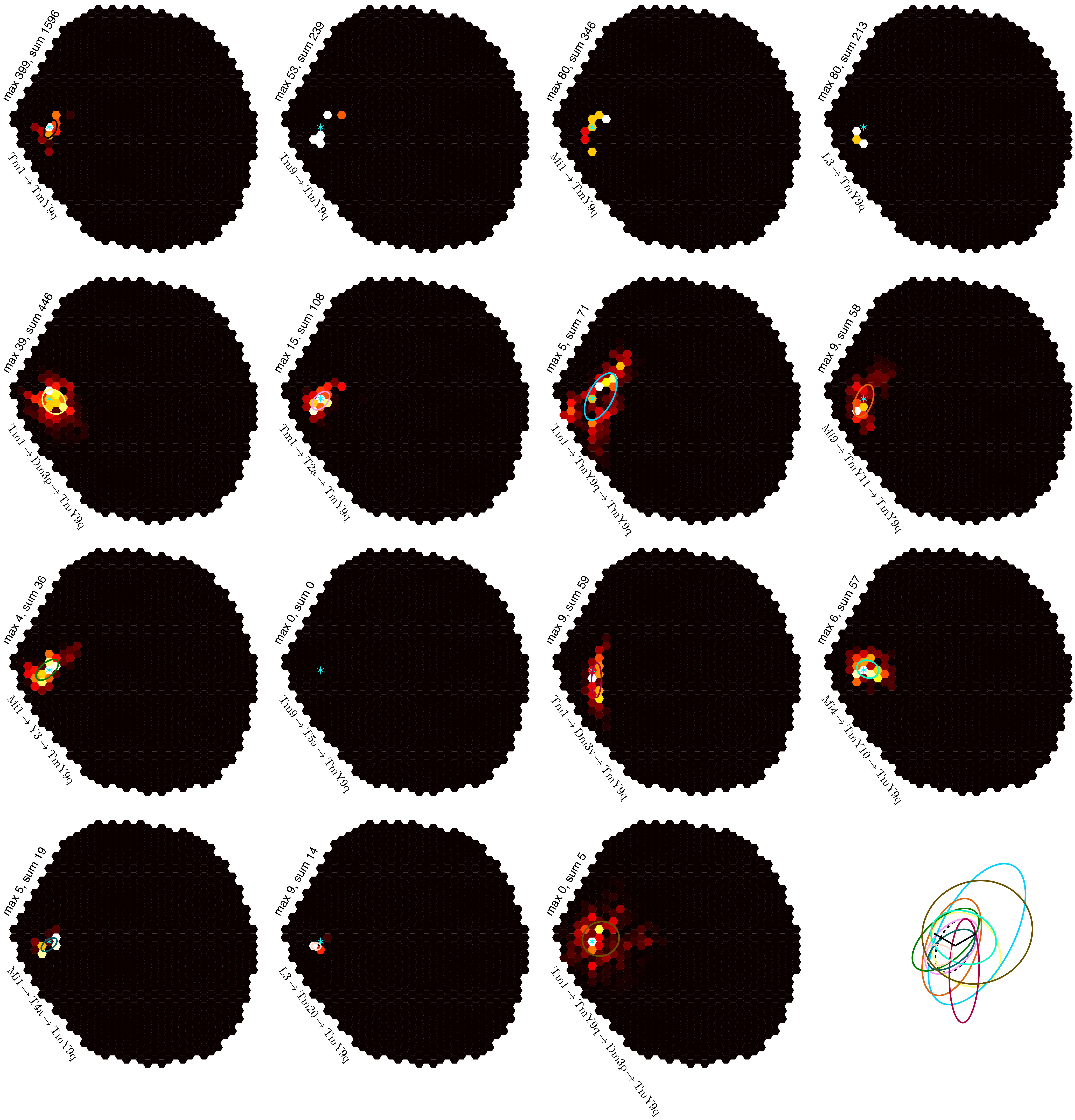
max 399, sum 1596
Tm1 → TmY9q
max 53, sum 239
Tm9 → TmY9q
max 80, sum 346
Mi1 → TmY9q
max 80, sum 213
L3 → TmY9q
max 39, sum 446
Tm1 → Dm3p → TmY9q
max 15, sum 108
Tm1 → T2a → TmY9q
max 5, sum 71
Tm1 → TmY9q → TmY9q
max 9, sum 58
Mi9 → TmY11 → TmY9q
max 4, sum 36
Mi1 → Y3 → TmY9q
max 0, sum 0
Tm9 → T5a → TmY9q
max 9, sum 59
Tm1 → Dm3v → TmY9q
max 6, sum 57
Mi4 → TmY10 → TmY9q
max 5, sum 19
Mi1 → T4a → TmY9q
max 9, sum 14
L3 → Tm20 → TmY9q
max 0, sum 5
Tm1 → TmY9q → Dm3p → TmY9q
